# Sexual and reproductive health service delivery innovations and adaptations during COVID-19: A systematic review and crowdsourcing open call

**DOI:** 10.1371/journal.pgph.0002032

**Published:** 2025-09-10

**Authors:** Eneyi E. Kpokiri, Megan L. Srinivas, Tigest Tamrat, Hayley Conyers, Heather L. Shams, Hannah Kerr, Nihal Said, Yusha Tao, Jennifer Bissram, Doruk Sahin, Roxanne Oroxom, Ulrika Rehnstrom Loi, Caron R. Kim, Bela Ganatra, Lale Say, Joseph D. Tucker

**Affiliations:** 1 Department of Clinical Research, Faculty of Infectious and Tropical Diseases, London School of Hygiene and Tropical Medicine, London, United Kingdom; 2 Institute of Global Health and Infectious Diseases, University of North Carolina, Chapel Hill, North Carolina, United States of America; 3 UNDP-UNFPA-UNICEF-WHO-World Bank Special Programme of Research, Development and Research Training in Human Reproduction (HRP), Department of Sexual and Reproductive Health and Research, World Health Organization, Geneva, Switzerland; 4 International Planned Parenthood Federation, IPPF, London, United Kingdom; 5 Social Entrepreneurship to Spur Health, SESH, Guangzhou, China; 6 Health Sciences Library, University of North Carolina, Chapel Hill, North Carolina, United States of America; PLOS: Public Library of Science, UNITED STATES OF AMERICA

## Abstract

This paper sought to identify and describe the innovations and adaptations implemented to ensure delivery of Sexual and Reproductive Health services during the COVID-19 pandemic and the potential for enhancing SRH services in other settings or in future emergencies. We searched five databases including PubMed, EMBASE, Scopus, Cochrane Library, and CINAHL. The review was registered on Prospero (CRD42022329411). The open call was launched and promoted widely; each submission was screened by five independent reviewers. The GRADE-CERQual methodology was used to assess confidence in each study finding. A thematic synthesis approach was employed for textual data and for studies with similar outcomes, a fixed effects model was employed. We identified 10,891 citations and 78 studies were included. We received 80 submissions to the open call, and 18 submissions contributed to the study findings. Submissions came from 42 countries, most of which were LMICs (37/42). Telemedicine was one main mode of continuing SRH services during the pandemic (moderate certainty). Teleabortion, or the provision of medication abortion remotely via telemedicine, was found to be a safe and effective way to maintain abortion service (97·9% of cases with 95% CI = 95·6 to 99·4%). However, increased reliance on telemedicine exacerbates inequities for low-income and rural populations. Self-care and self-testing enabled individuals to receive care for STIs (moderate certainty). This work identified strategies used to deliver SRH services during COVID-19 and the data suggest that many strategies relied on telemedicine to sustain SRH services. Self-care interventions were also used to sustain delivery of SRH services. There is need for further research to understand the long-term impact of these interventions and how they can be sustained over time.

## Introduction

The COVID-19 pandemic profoundly influenced the provision of sexual and reproductive health (SRH) services [1]. Essential SRH services were re-oriented, limited, or entirely stopped because of the pandemic [2]. The COVID-19 pandemic restricted travel to clinics, decreased the capacity of health professionals and disrupted essential supply chains for many essential diagnostics and medicines. As a result, population-based studies and other research suggest that many people had difficulty accessing SRH services during 2020–2022 [3]. While the COVID-19 pandemic undoubtedly presented many challenges, the pause in routine services also provided an opportunity to explore strategies and identify new ways to deliver SRH clinical services [4]. Several innovations, modifications and other health system responses were implemented to overcome the constraints on SRH services introduced by COVID-19 [1,5]. Documenting and analyzing the range of service delivery modifications is essential for sustainability and planning for subsequent pandemics and related emergencies.

In response to this gap, we conducted a systematic review to identify strategies that were available in the published literature. We complemented the systematic review with a global crowdsourcing open call to identify the health system response, service delivery adaptations, and innovations that have been implemented to continue SRH service provision during COVID-19. Employing a crowdsourcing approach ensured we could also identify implementations, strategies and experiences that may not have been already published and retrieved through the formal literature review, such as in approaches used in low- and middle-income countries (LMICs). This study describes findings from a qualitative evidence synthesis of data resulting from the systematic review and the global crowdsourcing open call.

## Methods

### Systematic review

PubMed, EMBASE, Scopus, Cochrane Library, and CINAHL were electronically searched on April 28, 2022, and for studies published between January 1, 2020, and this date. A trained public health librarian (JB) developed the strategy and conducted the search. An exhaustive list of search terms was used, combining the three constructs: SRH services, innovations and adaptations and COVID-19. Only articles with primary data and those published in English, Spanish, or French were considered for inclusion.

**Fig 1:**
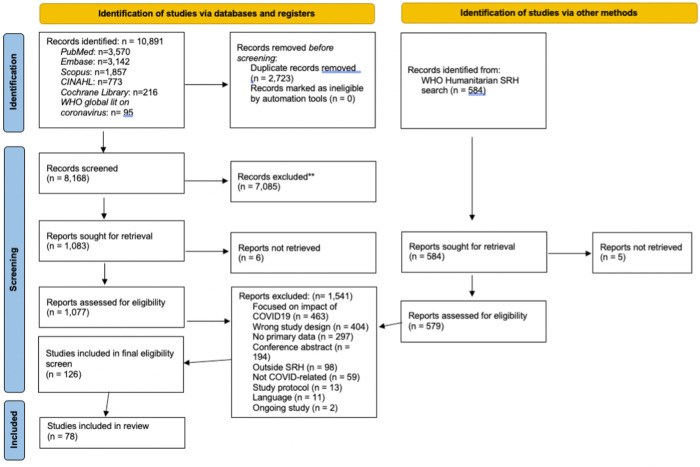
PRISMA flow diagram detailing study selection for the systematic review.

Titles, abstracts and full texts were reviewed by six team members (HC, MS, EK, HS, TT, and RO). Each title and abstract were reviewed by two team members independently, and conflicts were resolved by discussion to reach a mutual agreement. Non-duplicate articles identified from a parallel humanitarian SRH systematic review were also included at the full-text screening stage (Fig 1) [6]. Articles for full-text review were further screened by one reviewer (MS) to ensure that inclusion and exclusion criteria were uniformly applied.

We included articles describing strategies, innovations and modifications made for SRH service delivery during COVID-19, studies with primary data, and in English, French or Spanish language. All quantitative and qualitative study designs with primary data were considered for inclusion. We excluded reviews, commentaries and abstracts, articles describing innovations not SRH focused or not during the COVID-19 period. Studies that described health system responses or service delivery unrelated to the COVID-19 pandemic, those focused on adaptations outside of SRH, and articles including commentaries, expert opinions, and studies without primary data were excluded.

Data were extracted using Covidence systematic review software (version 2, Veritas Health Innovation, Melbourne, VIC, Australia). The following data were extracted: first author, publication year, publication location, study design and category, sample size, study setting, study population, country income (as designated by The World Bank) [7], descriptions [7] description of innovation or adaptation, COVID-19 related concern addressed, the outcome of the innovation/adaptation, barriers, and facilitators of the innovation/adaptation, strength and limitations of the study, justification for adaptability. Included articles were categorized based on the type of service delivery adaptation and/or health system response as well as SRH thematic areas. Where possible, we applied existing classification schemes to categorize the different interventions, such as the Classification of Digital Health Interventions and Self-care interventions [8,9].

Data extraction was conducted by two team members (HC and DS). Critical appraisal of the articles included for data extraction was conducted using the Critical Appraisal Skills Programme (CASP) tool by one analyst (HC) and reviewed by two team members (EK and MS).

### Crowdsourcing open call

The crowdsourcing open call employed the methodological guidance in the UNICEF/UNDP/World Bank/WHO Special Programme for Research and Training in Tropical Diseases (TDR) practical on crowdsourcing [10,11]. The open call was implemented in the following stages 1) convene an advisory team to finalise the call for submissions 2) promote and engage the public to contribute; 3) evaluate submissions received based on pre-specified criteria; 4) recognize finalists and 5) share the solutions with the broader public [10,11].

Once the details of the open call were finalised, a website was created to host the details of the call. Promotional banners were created with QR codes linked to the website. An official announcement was created and shared widely on social media platforms and disseminated through email lists of interest groups and in a health equity-focused in-person meeting hosted in Miami, FL by George Washington University’s Mullen Institute in Miami, FL.

The open call advisory group included clinicians, researchers, and policymakers. The advisory group met once weekly for a one-hour teleconference call to review details of the different stages of the open call. Updates to the open call were presented to WHO regional colleagues over a video conference meeting, and feedback was provided from the diverse group interested. For the open call we allowed submissions from all six [6] official language of the WHO including Arabic, Chinese, English, French, Spanish and Russian.

All submissions were screened by two independent reviewers (HC and HS) for eligibility using pre-specified criteria. A third reviewer (EK) was contacted to resolve submissions tied to eligibility votes from the initial two reviewers. All eligible entries were sent to volunteer judges for scoring using a standardized rubric and score sheet (S1 Table). The scoring rubric has 10 criteria points, and a submission will have to score the maximum 1 point across the 10 criteria to reach the full mark of 10 points. This submission typically will provide a very clear description of the change/ adaptation or innovation and should be easy to understand by a non -expert. Volunteer judges were recruited based on their experience in SRH research and practice. Reviewers connected to any submissions were asked to recuse themselves for reviewing that entry. Three independent judges reviewed each submission using the pre-designed judging rubric (S1 Table), and all judges provided a score of 1–10 for each submission assigned to them. The mean scores and standard deviations (SD) were calculated. Submissions with SD greater than 2 were sent to an additional independent reviewer. Submissions that achieved a mean score of 8 or greater out of 10 were selected as finalists’ submissions. Top ranked ideas within the finalists’ group were also invited to write up final reports and considered for co-authors in resulting publications.

### Data analysis

We employed a parallel mixed methods approach to analyse and present the data. The quantitative data, mainly participant and submission characteristics, was analysed and presented using basic descriptive frequencies. The qualitative data was analysed thematically using the framework approach, including familiarization with the data, coding, charting, and mapping out themes for interpretation [12,13]. In terms of validity and reliability in presenting findings, the eligible submissions were coded separately by two independent reviewers, and the emerging codes and themes were further reviewed and synchronised by a third team member. An adapted Critical Appraisal Skills Program Checklist (CASP) was used for quality appraisal of the submissions [14] and then the GRADE-CERQual (Confidence in the Evidence from Reviews of Qualitative research) method was employed to rate the confidence level of the key thematic findings.

If at least two studies focused on a certain SRH intervention, then a pooled analysis was performed. The proportion of patients for each outcome was pooled using random-effects meta-analysis (meta prop in Stata), with 95% confidence intervals (CIs) based on the exact binomial method. We examined statistical heterogeneity between studies using the I^2^ statistic. Analyses were performed in Stata version 16.1 (Stata Corp, College Station, Texas, USA).

### The GRADE CERQual approach

We employed the Grading of Recommendations Assessment, Development, and Evaluation Confidence in the Evidence from Reviews of Qualitative Research (GRADE-CERQual) approach [15,16]. This approach evaluates the study findings on 4-point criteria, including methodological limitations, coherence, adequacy and relevance of the studies in this case submissions supporting a review finding, the relevance of included studies to the research aim, the coherence of the review finding, and the adequacy of the data informing the findings (S2 Table) [17,18].

### Ethical consideration

The protocol (A66032) was developed by the team and approved by the WHO/HRP Research Review Panel and the WHO Ethics Review Committee (WHO ERC) with approval number CERC.0176. The systematic literature review was registered on Prospero (CRD42022329411) and reported according to PRISMA guidelines [19] and the protocol is available on Protocols.io [20]. All finalists from the crowdsourcing call had provided consent to share and disseminate their contributions.

## Results

### Systematic review

The systematic review search identified 10,891 citations for screening. Of those, 78 studies (S3 Table) met the criteria to be included for data extraction and analysis (Fig 1). Studies included were based in various settings, featuring diversity in geography, country income status, and focus populations (Table 1). Data were from 78 countries, including 63 high-income (HICs), 12 middle-income (MICs), and one low-income countries (LICs). There were also two studies that were multi-country analyses (breakdown of country demographics for these studies is included in Table 1). 48 studies focused on telemedicine approaches, eight on self-care interventions and seven on community-based approaches. Of the telemedicine approaches, six studies focused on telemedicine approaches to delivering medical abortion care presented quantitative data examining these services’ efficacy, safety, and patient satisfaction. Three primary outcomes were evaluated: safe medical abortion rate (the number of people experiencing uncomplicated medical abortion divided by the number of people who were linked to telemedicine approaches to delivering medical abortion care); adverse events after abortion (the number of people who had adverse events after abortion among those who received medical abortion care through telemedicine approaches), and satisfaction with the telemedicine medical abortion care using a (the number of people who were “very satisfied” or “satisfied” with the medical abortion care through telemedicine approaches services divided by the number of people who have received the services.

**Table 1:** Characteristics of studies from the systematic review and submissions included from the open call.

Number of Articles	N = 148	Crowdsourcing data (n = 70)	Systematic Rev. (n = 78)	n% (articles)
Country income levels***				
	High	8	63	49%
	Upper-middle	17	5	15%
	Lower-middle	31	7	26%
	Low	20	1	14%
Multi-country (21 countries from LMICs) *	42.86% low, 52.38% lower-middle, 4.76% upper-middle		1	
Multi-country (71 countries from all around the Globe) **	10% low, 64% middle, 26% high		1	
Setting***				
	Urban	23	48	48%
	Suburban/peri-urban	17	14	21%
	Rural	22	7	20%
	Mixed	21	12	22%
	Not defined	6	14	14%
**Study Category**				
	Quantitative		47	60%
	Qualitative		9	12%
	Mixed methods		22	28%
SRH Focus Area***				
	Antenatal care	18	24	28%
	Contraception counselling and provision	26	10	24%
	Fertility care	4	–	3%
	GBV	23	2	17%
	General gynaecological care	–	3	2%
	HIV and STIs	28	24	35%
	Information and education	44	5	33%
	Intrapartum care	9	–	6%
	Oncology	–	4	3%
	Post-natal care	13	10	16%
	Safe abortion care	8	14	15%
	Sexual function	6	–	4%
**Population of focus**				
	Adolescents	23	5	6%
	Cancer patients	–	3	4%
	General adults	21	3	4%
	Health professionals	–	12	15%
	LGBTQ/MSM	2	4	5%
	PLWH	–	9	12%
	Pregnant individuals	–	15	19%
	Women	19	28	36%
	Children	2	2	3%
	Other****	3	2	3%

*Countries include (Burundi, Cote d’Ivoire, Democratic Republic of the Congo (DRC), Dominican Republic, ESwatini Ethiopia, Kenya, Lesotho, Malawi, Mozambique, Uganda, Zambia, Zimbabwe, Haiti, South Sudan, Tanzania, Angola, Cameroon, Nigeria, Rwanda, Vietnam).

**The article does not include the full country listing.

***Includes crowdsourcing data, (n = 70).

****Other: female sex workers (n = 1), indigenous people (n = 1).

### Open call submissions

We received a total of 80 submissions, of which 70 were submissions from individuals and organizations eligible for judging. Thirteen submissions had a mean score of 8 or greater out of 10 (S4 Table). The open call steering committee had chosen 8 to be a benchmark score to select exceptional or finalists’ submissions. Submissions were received from individuals and institutions from 42 unique countries, including five HICs, 26 LMICs, and 11 LICs. In terms of format, we received submissions as texts (n = 48), audio/video (n = 6), and a combination of text/picture or infographic (n = 9). For languages, we received submissions in English (n = 48), French (n = 4), and Spanish (n = 3).

Most of the innovations had been implemented and sustained (84%) and a few had already been discontinued (16%). With regards to setting/ level of care, 46 submissions (34%) were implemented either in the home or other settings within the community. Forty-one entries (30%) were implemented at the primary care level, 21 entries (16%) in secondary care, and 13 entries (10%) were implemented in tertiary care.

Submissions and studies targeted one or more SRH focus themes (Table 1), including antenatal care (50%), sexual health information and education (57%), prevention and control of HIV and other STIs (35%), contraception and family planning (24%), fertility care and sexual function (31%), gender-based violence (17%), and comprehensive abortion care (15%). Populations included in the submissions include women (57%), adolescents (6%), children (3%), adults and the general population (4%). Key populations of focus included people living with HIV/AIDS (PLWH) (12%) and sexual minorities (5%).

## Facilitators in the delivery of SRH services during COVID-19

### Telemedicine

Forty-eight studies and nine submissions described the use of telemedicine for the provision of healthcare services during COVID-19 (moderate certainty of evidence, Table 2) [21,22]. Use of virtual clinic as a form of telemedicine was commonly employed to sustain access to medical services without going to facilities. Virtual clinics here refers to virtually scheduled appointments between healthcare providers and patients for delivery of health services. In total, 63 studies and eight submissions focused on higher-income individuals and 48 studies and 23 submissions focused on urban populations local health care workers (HCWs). The HCWs also had improved access to specialists in their field via live teleconsultations for advice regarding specific issues. Hotlines were implemented for SRH and Gender Based Violence (GBV) advice, and unstructured supplementary service data (USSD) messaging services were created for general advice and counselling on SRH. SRH communication used text, online videos, radio messages, USSD and other mobile phone applications. Perinatal counselling and care, such as breastfeeding video tutorials, also shifted to electronic platforms to deliver care while reducing exposures for high-risk patient populations.

**Table 2:** Summary of findings from the open submissions and systematic review studies GRADE CERQual Assessments.

Facilitators to accessing SRH services during COVID-19 from the open call and systematic review						
Study finding	Studies and submissions contributing to the finding	Methodological limitations	Coherence	Adequacy	Relevance	CERQual Assessment
*Telemedicine: This was used as a key strategy to increase access to SRH during COVID. This included telephone, video conference and other online app-based appointments.	Studies: (3-5) (7-10) (15-17) (20) (22) (24) (25) (26) (28-31) (33) (35-37) (39) (43-46) (49-50) (52) (54) (56) (58-66) (68-70) (72-75) (77)	Serious	Minor	Minor	Minor	
	Submissions: (1) (2) (3) (4) (5) (6) (9) (10) (14)	Moderate methodological concerns	Moderate concerns – mixed data presented	Minor concerns – significant and rich qualitative data	Minor concerns – all strategies highly relevant	Moderate
**Teleabortion:** This is medical abortion using telemedicine and was used to continue service delivery and increased privacy for patients seeking care including services which continued despite travel-related restrictions.	Studies: (3) (9) (10) (24) (26) (30) (44) (46) (49) (52) (65) (78)	Moderate	Minor – consistent findings	Minor	Minor	
	Submissions (1) (2) (15)	Serious concerns – limited methodological information.	Minor concerns- consistent findings	Moderate concerns, limited number of strategies	Minor concerns –	Moderate
**Self-care and decentralization: i**ncluding self-testing and self-sampling empowered individuals to address their own SRH needs during lockdowns. STI/HIV testing at home as well as self-applied tools to monitor conditions such as pre-eclampsia were enhanced.	Studies: (14) (19) (21) (23) (32) (42) (55) (67)	Moderate	Moderate – some mixed data	Minor	Minor	
	Submissions (1) (2) (3) (13) (18)	Moderate - Limited methodologies reported	Minor concerns – no opposing data noted.	Serious concerns – based on secondary to review findings	Minor - highly relevant to COVID-19	Moderate
**Community based services**: healthcare workers, lay staff and other community services was utilized to drive delivery of SRH services during COVID. This included distribution of HIV testing kits, ARTs, OTC, and other prevention services which lead to increased medication (treatment and PrEP) utilization.	Studies: (12) (38) (42) (48) (53) (57) (71)	Serious – limited methodologies reported	Minor	Moderate	Minor	
	Submissions: (3) (4) (5) (6) (7) (8) (10) (11) (12) (16)	Moderate - limited methods in three strategies	Minor concerns – no opposing data noted.	Minor concerns – Variety of data from LMICs and HICs	Minor concerns - highly relevant to SRH and COVID-19	Low

*Telemedicine is defined as the utilization of electronic information and telecommunications technologies to remotely offer medical and healthcare services. This was one of the main modes of continuing SRH services despite pandemic restrictions.

**Fig 2:**
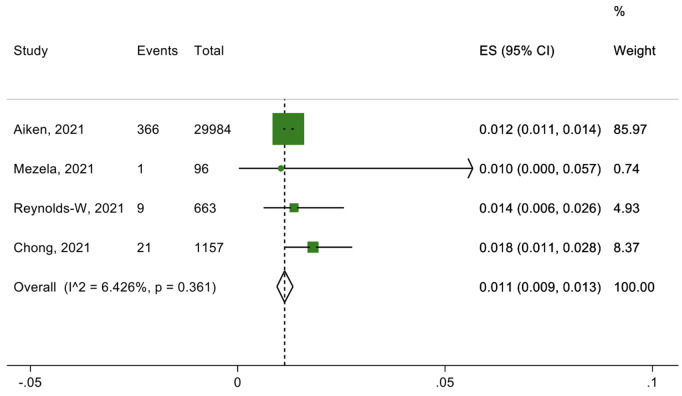
Percentage of unsuccessful medical abortions.

**Fig 3:**
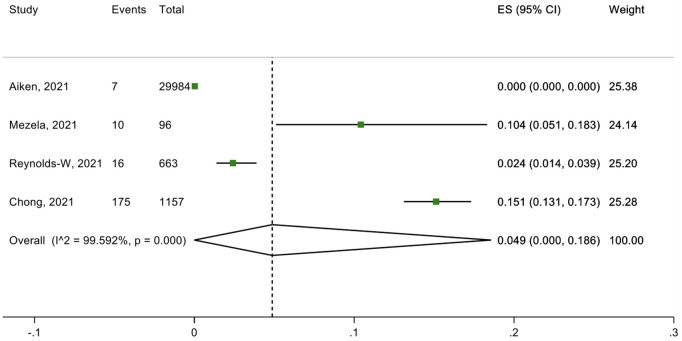
Safety (percentage of adverse events after abortion).

**Fig 4:**
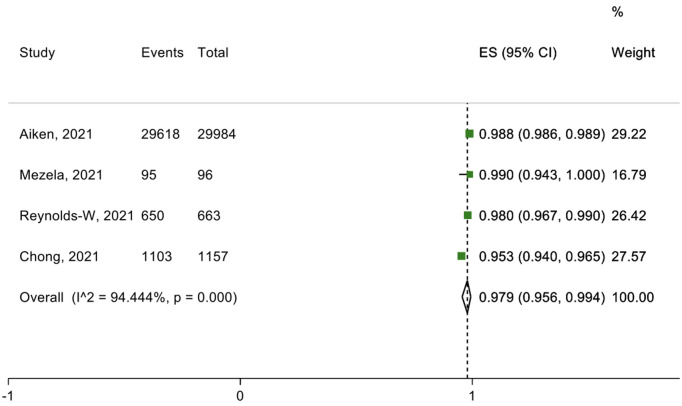
Percentage of successful medical abortions.

Eleven studies and three submissions discussed telemedicine approaches to delivering medical abortion care (teleabortion). One submitted approach included an online consultation with an HCW, after which the patient is sent a kit with the medication, short-term contraception, and information to facilitate self-managed abortion. Of the eleven studies on medical abortion through telemedicine, six studies specifically looked at the outcomes of efficacy, safety, and patient satisfaction (Figs 2–4).

The six studies out of the 11 presented quantitative data on telemedicine approaches to delivering medical abortion care assessed the services’ efficacy, safety and patient satisfaction. Three primary outcomes were evaluated: successful medical abortion (defined as uterine emptying by medication without the need for surgical intervention), adverse events after abortion (e.g., need for hospitalization, bleeding needing a blood transfusion or additional procedures), and satisfaction with the medical abortion through telemedicine (rating “very satisfied” or “satisfied”). A total of 97·9% (95·6-99·4%) of those who received telemedicine services had experienced successful medical abortions, and 1·1% (0·9-1·3%) had experienced unsuccessful medical abortions (Figs 2 and 3). In total, 4·9% (0-18·6%) of those who received medical abortion care through telemedicine that experienced adverse events after the abortion (Fig 4). Regarding the acceptability towards telemedicine approaches to delivering medical abortion care in the future, 97·4% (95·4-98·9%) of those were “very satisfied” or “satisfied” with this service, and 80·0% (71·3-87·4%).

### Self-care interventions

Eight studies and five submissions described self-care interventions during COVID-19 for HIV and STIs (moderate certainty of evidence, Table 2). Self-care is the ability of individuals, families and communities to promote their own health, prevent disease, maintain health, and to cope with illness and disability with or without the support of a health worker [8]. This included HIV self-testing, syphilis self-testing, and HPV self-collection.

### Community-based strategies

Seven studies and ten submissions focused on using community-based resources to improve SRH service delivery during COVID-19. Community-based peer educators were leveraged to deliver sexual education services which was needed to meet the increased demand for SRH services during the COVID-19. Peer education also played a major role, particularly among adolescents and young adults.

Community Health Workers (CHWs) visited people at home to support the delivery of SRH services during COVID to support self-care interventions. Other examples included pharmacies delivering medication directly to patients to limit individual travel. Specifically mentioned were anti-retroviral medications, oral contraceptives, and medications for abortion. One-stop shops were created to minimise repeated visits by CHWs or in-person attendances for antenatal and postnatal care services. These shops are strategically located within the communities providing a range of SRH services, including testing services, counselling, and drug refill, e.g., oral contraceptives and anti-retroviral drugs. Seven interventions also recruited lay community members to disseminate information and recruit new people to utilize HIV pre-exposure prophylaxis (PrEP) and at-home testing.

## Discussion

We synthesized data from a systematic review and crowdsourcing open call to identify innovative SRH solutions during COVID-19. Many service providers adapted existing interventions in their local settings, and others implemented entirely new innovations. This study expands the literature by aggregating data from a systematic review and an open call, focusing on innovations in response to COVID-19 and examining medical abortion care through telemedicine services. Prior reviews demonstrated the decline in SRH care during the pandemic, suggesting the need for innovation [4,23].

The analysis of the pooled data from these studies demonstrated that telemedicine approaches tele-abortion were not just feasible but a safe and effective means of providing medical abortion services during the pandemic. This is consistent with the literature on telemedicine approaches to delivering medical abortion care [24]. Medical abortion care through telemedicine increased the ability for many individuals to receive timely and confidential care earlier in pregnancy than reliance on only in-person care. Successful adaptations such as this should continue beyond the need for COVID-19 service alterations [25].

While some types of care benefited from remote models of delivery, patients with more complex medical issues, such as oncological conditions, noted the difficulty in receiving comprehensive care through such platforms [26–28]. Our data also show that health inequities can be exacerbated with reliance on telemedicine-only adaptations. Poor internet access in rural areas and amongst low-income populations created further inequities in those able to receive SRH services during the pandemic, especially as many transitioned to telemedicine [27,29–33]. The majority of studies found low bandwidth and unstable internet access a common challenge for adaptations that used telemedicine for continuing SRH care. Many low-income and rural populations with less internet access could not use telemedicine. One study used community health workers with technology access such as smart phone or internet enabled devices to help overcome some of these inequities, demonstrating a possible solution to expand the reach of telemedicine adaptations [33].

Several community-based and self-care interventions was used to improved access to services during the COVID-19 lockdowns. Overall, our findings demonstrate that no single approach is sufficient to overcome the pandemic-based challenges to SRH delivery. Instead, a collaborative approach implementing a hybrid of modalities best addresses SRH service needs. This is consistent with a sister scoping review essential services for maternal, newborn, child, and adolescent health during the COVID-19 pandemic [34].

This study has several limitations. First, the search attempted to encompass all innovations in service delivery, making the findings quite broad in nature. The systematic review included several digital terms, which resulted in a heavy telemedicine presence. Additionally, only studies with primary data were included to screen for quality. However, this did result in the exclusion of some high-quality interventions still in early stages of implementation. Additionally, this data requirement alongside rapid publication led to significantly higher representation of HICs in the systematic review. The crowdsourcing arm of this study attempted did not have primary data or publication requirements, which helped to overcome these latter limitations in the overall findings. Additionally, the open call process, which did not have predetermined categories, also yielded similar results with the review, reinforcing our findings. Also, we did not collect the data in health system levels at which the innovations were initiated, as this would have been helpful to know at level of the health system and at whose initiatives some of these innovations were introduced. Finally, the date of the search was April 28 2022 which was before WHO had declared the pandemic to be over.

This work has several implications for research, policies, and practice. From a research perspective, further implementation research is needed to determine how innovations from the COVID-19 period can be sustained as restrictions are relaxed. Several innovations developed in response to COVID-19 may need to be adapted in order to align with non-COVID-19 health systems and programs. From a policy perspective, our data provide robust evidence to support tele-abortion services. From a program perspective, we need more data on the use of telemedicine among specific subgroups (poor people, rural people) to ensure that this approach can expand SRH access [35].

In summary, this study demonstrated that several strategies were leveraged to continue the provision of SRH services during COVID-19. These include telemedicine, self-care interventions and community-based strategies. This work expands our understanding of providing care during emergencies and lockdown situations. However, there is need for more research to how these strategies identified strategies can be adapted and implemented in diverse settings to help overcome impediments to SRH service delivery.

## Supporting information

S1 Table Judging rubric for scoring open call submissions. (DOCX)

S2 Table Components of GRADE-CERQual assessments. (DOCX)

S3 Table Included systematic review studies. (DOCX)

S4 Table Open call submissions contributing to the study findings. (DOCX)
