# Supplementary material for: Sexual and reproductive health service delivery innovations and adaptations during COVID-19: A systematic review and crowdsourcing open call
Source: PLOS Glob Public Health. 2025 Sep 10;5(9):e0002032. doi: 10.1371/journal.pgph.0002032 (PMC12422507; doi:10.1371/journal.pgph.0002032)
Supplement: S1 Table — (DOCX) [file pgph.0002032.s001.docx]

**S1 Table: Judging rubric for scoring open call submissions**

| **Criteria/Score (1-10)** | **1-3** | **4-6** | **7-10** |
| --- | --- | --- | --- |
| **A) Clarity of the change or adaptation and innovation** | | | |
| 1)Does this submission provide a **clear description** of a change, adaptation or innovation within the field of SRH?  Does the entry present a concept that is easy to understand stand-alone (i.e. without seeking further information)? | The exact change, adaptation or innovation has not been clearly described | The ideas presented in this submission are well described | This entry provides a very clear description of the change/ adaptation or innovation and easy to understand |
| 2)Does this entry provide **quality account of innovative new ideas or concepts** that are not already within existing service delivery or commonly  used in practice? | This entry highlights ideas and concepts that apply to already existing SRH service delivery services. | This entry provides some unique and creative insights relating to adaptations for SRH  services | This entry provides a new and unique take on sustaining delivery of SRH services during  Covid-19. |
| **B) Feasibility and adaptability** | | | |
| 3) Is the entry **feasible** and suitable in the local context where it was implemented or used? | This entry provides little content or considerations for effective service delivery in the local setting where it was implemented | This entry can demonstrate some level of feasibility and suitability in the settings/context where it was implemented | This entry demonstrates a high level of feasibility, and it is suitable in the settings where it was implemented. |
| 4) Does the submission offer potential to be **adapted** for use in other settings different from where it was originally implemented? | This entry has little to no potential for adaptation and use in settings outside where it was originally developed/ implemented | This entry provides some potential for adaptation and use in settings outside where it  was originally developed/ implemented | This entry clearly shows the potential to be adapted and easily used in different settings |
| 5) Has this entry provided considerations for **scalability** and wider implementation? | This entry provides little, or no considerations related to scalability and wider use for the delivery of SRH services. | Provides some relevant considerations related to scalability and wider use for the delivery of SRH services. | This entry clearly describes relevant, innovative, and useful concepts and ideas for  scaling up implemtation |
| 6) Does this entry demonstrate **sustainability** in the long term and that it can continue effective delivery of SRH services even beyond Covid-19 and in the event of future disruptions? | This entry provides little or no ideas for sustained and effective delivery of SRH services beyond Covid-19 and in the event of future crisis | This entry provides some relevant considerations related to sustaining the delivery of SRH services in crisis | This entry contains robust content and provides relevant, innovative, and useful concepts and ideas for sustained and effective delivery of SRH  services |
| **C) Impact and potential for use in other settings** | | | |
| 7) Are the **impact** and outcomes of this innovation in SRH service delivery reported in the entry? | This entry does not report on any impact, or the potential outcomes expected | This entry provides some descriptions of impact or expected outcomes with the  change | Impact following service the modification is clearly described in this entry |
| 8) Does this entry provide considerations and implications for **gender, rights, and equity**? | This entry does not provide any considerations and/or implications for gender, rights and equity | Provides some relevant considerations and implications for gender, rights and equity | Provides detailed considerations and implications for gender, rights and equity are included |
