# Supplementary material for: Sexual and reproductive health service delivery innovations and adaptations during COVID-19: A systematic review and crowdsourcing open call
Source: PLOS Glob Public Health. 2025 Sep 10;5(9):e0002032. doi: 10.1371/journal.pgph.0002032 (PMC12422507; doi:10.1371/journal.pgph.0002032)
Supplement: S2 Table — (DOCX) [file pgph.0002032.s002.docx]

**S2 Table: Components of GRADE-CERQual assessments**

| **Component** | **Definition** |
| --- | --- |
| Methodological  limitations | The extent to which problems were identified in the conduct of the primary  studies that contributed to the evidence for a review finding |
| Relevance | The extent to which the primary studies supporting a review finding are  applicable to the context specified in the review question |
| Coherence | The extent to which a review finding is based on a pattern of data that is similar  across multiple individual studies and/or incorporates (compelling) explanations for any variations across individual studies |
| Adequacy of data | An overall determination of the degree of richness and/or scope of the evidence  and of the quantity of data supporting a review finding |
