# Supplementary material for: Sexual and reproductive health service delivery innovations and adaptations during COVID-19: A systematic review and crowdsourcing open call
Source: PLOS Glob Public Health. 2025 Sep 10;5(9):e0002032. doi: 10.1371/journal.pgph.0002032 (PMC12422507; doi:10.1371/journal.pgph.0002032)
Supplement: S3 Table — (DOCX) [file pgph.0002032.s003.docx]

**S3 Table: Included systematic review studies**

| **Author** | **Description** |
| --- | --- |
| 2 Adams, S.J. et al. | Remote ultrasound examination for pregnant women in an indigenous community with a telerobotic arm. |
| 3 Aiken, A. R. A. et al. | Hybrid tele-abortion service provision model for pregnant women in their first trimester. |
| 4 Aksoy, D. Y. et al. | Tele-education program for pregnant women. |
| 5 Arias, M.P. et al. | Postpartum care conducted through teleconsultations. |
| 6 Bailey, L. E. et al. | Country policy analysis of increased MMD-spans for antiretroviral drug provision and its indications for viral suppression. |
| 7 Beatty, K. E. et al. | Analysis of key policies for the possibility of telehealth for contraceptive care. |
| 8 Bock, M. J. et al. | Virtual video-based home visits in perinatal and early childhood home visiting. |
| 9 Boydell, N. et al. | Telemedicinal abortion service implemented in response to the coronavirus (COVID-19) pandemic. |
| 10 Brandell, K. et al. | Telemedicinal abortion through WoW (Women on Web), a web-based fully remote tele-abortion service. |
| 11 Campbell, B.R. et al. | Behavioral changes in PLWH on an in-app patient-provider messaging platform. |
| 12 Boyd, A.T. et al. | Community-based, rather than facility-based, HIV case-finding; immediate initiation of newly- diagnosed PLHIV on 3-month ART starter packs (first ART dispense of 3 months of ART) along with expansion of ART distribution through community refill sites supplemented by targeted client bulk messaging for refill reminders, and broadened access to multi-month dispensing (MMD) (3–6 months ART) among PLHIV established in care. |
| 13 Campbell, K. J. et al. | Shift toward the immediate in-facility PNC provision to women after birth. |
| 14 Carnevale, C et al. | Home delivery of gonorrhea/chlamydia testing kits for HIV prevention patients accompanied by a telehealth video consultation. |
| 15 Cheng, Y. et al. | Outline of telehealth practices both remote and hybrid for sexual and reproductive health. |
| 16 Jeganathan, S. et al. | Utilization of telehealth practices for the delivery of high-risk obstetrical care in a large healthcare system. |
| 17 Limaye, M. A. et al. | Utilization of telehealth practices for prenatal obstetrical care. |
| 18 Chen, M. et al. | Country-wide implementation of an asynchronous online telehealth platform for teleconsultations for obstetrical care. |
| 19 Hoagland, B. et al. | Implementation of home-delivered HIV self-testing kits accompanied by teleconsultations. |
| 20 Galle, A. et al. | Implementation of telemedicinal practices including teleconsultations, provider-to-client education and healthcare provider communication, to maintain the provision of maternal and newborn healthcare during the COVID-19 pandemic. |
| 21 Fistonich, G. M. et al. | Provision of newly designed mail-in HIV and sexually transmitted infection testing kits. |
| 22 Duryea, E. L. et al. | Implementation of a hybrid teleconsultation model incorporating audio-only prenatal care visits. |
| 23 O’Byrne, P. et al. | Implementation of mail-in HIV self-testing kits. |
| 24 Porter Erlank, C. et al. | Implementation of early medical tele-abortion practices. |
| 25 Boshara, A. I. et al. | Implementation of teleconsultations for medical HIV-care visits. |
| 26 Tschann, M. et al. | Implementation of tele-abortion practices accompanied by at-home low-sensitivity pregnancy tests and/or patient symptom checklists for medication abortion follow-ups. |
| 27 Giannakoulis, V. G. et al. | Implementation of hybrid teleconsultation model in pediatric and adolescent gynecological care. |
| 28 Gloston, G. F. et al. | **Reinterpretation of video-based telehealth for HIV care, with new adaptations such as the introduction of organizational relations between external (research) and internal (clinical) staff, full implementation of telehealth modalities instead of a hybrid model.** |
| 29 Potenza, S. et al. | **Implementation of telehealth consultations for prenatal care.** |
| 30 Godfrey, E. M. et al. | **Asynchronous online consultations and mail-in abortion medication by family physicians through the online service "Aid Access".** |
| 31 Galaviz, K. I. et al. | **Implementation of audio-only teleconsultations for HIV Care and changes in provision of medication towards mail-in services in order to reduce physical contact within the context of COVID-19 regulations.** |
| 32 Ngu, S.-F. et al. | **Implementation of various outreach accompanied by education programs on HPV and self-sampling kits for cervical cancer screening.** |
| 33 Harsono, D. et al. | **Provision of appointments through the Epic-based MyChart patient portal and direct patient contact through clinical staff to schedule telemedicinal visits, which were in turn either conducted directly by audio-only phone calls or platform-based video conference links.** |
| 34 Phiri, M. M. et al. | **Implementation of a unique community-based framework called "Yathu Yathu", meaning "for us, by us", which includes provision of CSE sessions via Facebook and WhatsApp, establishment of spaces (hubs) located within the community away from the government-run health facility in each of the ten intervention zones and (ii) provision a prevention points card (PPC) as a form of incentivization and health record tracking modality.** |
| 35 Tseng, E. et al. | **Shift towards remote computer security services for victims of intimate partner violence.** |
| 36 Tozour, J. N. et al. | **Implementation of video-only teleconsultation for obstetrical care in an urban environment.** |
| 37 Stifani, B. M. et al. | **Implementation of both hybrid and remote teleconsultation models for contraceptive counseling and provision.** |
| 38 Pichon, L. C. et al. | **Digitalization of Whole YOUniversity, a series of educational training sessions on culturally congruent topics for LGBTQ individuals living in the U.S. South, during COVID-19 through online attendance, accompanied by face-to-face meetings.** |
| 39 Palmer, K. R. et al. | **Implementation of teleconsultations in antenatal care supplemented with a suite of patient and staff information sheets, and systems to support remote blood pressure checks and fetal growth assessments.** |
| 40 Hirani, S. A. A. et al. | **Design and creation of a knowledge mobilization tool; an animated video on “Breastfeeding during COVID-19”, and its dissemination through social media.** |
| 41 Wujcik, D. et al. | **Implementation of an electronic patient symptom management program which involves patient self- monitoring and symptom reporting through a digital platform.** |
| 42 Eustaquio, P. C. et al. | **Implementation of a community led online-based HIV self-testing demonstration project.** |
| 43 Rivera, A. et al. | **Implementation of HIV self-testing accompanied with video-based teleconsultation as an alternative to in-person HIV.** |
| 44 Reynolds-Wright,  J.J. et al. | **Implementation of tele-abortion with mail-in self-testing components.** |
| 45 Sonagli, M. et al. | **Enabling telemedicinal outpatient appointments through two security certified digital platforms. (Conexa Saúde®, Sul América Saúde®)** |
| 46 Godfrey, E. M. et al. | **Introduction of tele-abortion practices with components of intra-healthcare provider communication and provider-to-provider training.** |
| 47 Lim, A. C. et al. | **Adaptations made to a urban youth sexual health clinic in Florida that allowed uninterrupted provision of services while protecting against spread of COVID-19, that involves the utilization of PDSA (Plan, Do, Study, Act) cycles as a decision-making modality, which involved introduction of targeted client communication, on demand information services to client innovations and digitalization of client education programs.** |
| 48 Middleton, M. et al. | **Implementation of Test@Work, a project comprising text messaging to raise awareness of HIV along with follow up messaging targeting construction workers.** |
| 49 Mezela, I. et al. | **Introduction of a hybrid early medical tele-abortion model.** |
| 50 Marshall, J. et al. | **Introduction of virtual perinatal home visiting to the Maternal, Infant, and Early Childhood Home Visiting (MIECHV) program.** |
| 51 Sapire, R. et al. | **Analysis of state policies surrounding COVID-19 in relation to gender-based violence.** |
| 52 Upadhyay, U. D. et al. | **Introduction of a no-test tele-abortion services model.** |
| 53 Matambanadzo, P. et al. | **Adaptations made to the Sisters with a Voice (Sisters), a community-led differentiated HIV prevention and treatment services model that includes the education of client-peers through providers, health record tracking, along with establishment of digital communication modalities for peer educators.** |
| 54 Morgan, A. et al. | **Implementation of telehealth consultations for prenatal care services.** |
| 55 Moulaei, K. et al. | **Development of a mobile-based application to facilitate self-care for pregnant women.** |
| 56 Moyo, J. et al. | **Provision of teleconsultations for gynecological and obstetrical care via messaging on WhatsApp or SMS.** |
| 57 Mpofu, M. et al. | **Implementation of out-of-facility individual differentiated service delivery models for distribution of ART via private pharmacies and home delivery with couriers.** |
| 58 Mulhall, J. et al. | **Introduction of virtual oncological patient follow up via phone call teleconsultations.** |
| 59 Nakagawa, K. et al. | **Introduction of teleconsultations for antenatal care with a mailed in cardiotocograph and a sphygmomanometer.** |
| 60 Nakagawa, K. et al. | **Introduction of teleconsultations for antenatal care with a mailed in cardiotocograph and a sphygmomanometer.** |
| 61 Paul, J. J. et al. | **Various adaptions made to perinatal mental health group programming in order to adjust to telehealth circumstances, including on-site virtual peer-groups, consultation and provider to provider education.** |
| 62 Peahl, A. F. et al. | **Implementation of a prenatal care model incorporating reduced frequency visits and virtual visits in response to the pandemic.** |
| 63 Quinn, L. M. et al. | **Implementation of junior obstetric team virtual consultations for antenatal care supplemented by provider-to-provider communication to assist in diagnostic assessment and health prescriptions.** |
| 64 Reisinger-Kindle,  K. et al. | **Implementation of teleconsultations for antenatal and post-natal care, accompanied by digital healthcare provider communication and provider-to-provider training.** |
| 65 Reynolds-Wright,  J.J. et al. | **Implementation of a tele-abortion service model supplemented by on demand information services incorporating audiovisual resources on what to expect during the consultation.** |
| 66 Rodler, S. et al. | **Shift towards a virtual management of uro-oncological patients, consisting of virtual boards for providers to discuss diagnostics along with teleconsultations with patients.** |
| 67 Rosadiño, J. D. T. et al. | **Implementation of unassisted HIV self-testing supported by community-based client communication programs.** |
| 68 Rousseau, A. et al. | **Shift in policy of service provision regulations, in that midwives are allowed to conduct teleconsultations in perinatal health care.** |
| 69 Rybińska, A. et al. | **Protocol modification to Family Connects, a systems approach to supporting families, which consists of a virtualized evidence based postpartum home-visiting intervention.** |
| 70 Schwartz, B. N. et al. | **Addition of a fetal telemedicine intervention to fetal telecardiology and its expansion during the COVID-19 pandemic.** |
| 71 Shaikh, I. et al. | **Introduction of an hybrid telemedicine-accompaniment model based on a web-based app (SK - Sehat Karani) that makes telehealth SRHR services available to women and girls by using community- based health workers with access to smart devices as intermediaries between women and doctors.** |
| 72 Shields, A. D. et al. | **Conversion of a traditional MFM site to a telemedicinal model.** |
| 73 Stifani, B. M. et al. | **Implementation of teleconsultations to screen for on-site suitability of patients.** |
| 74 Wood, S. M. et al. | **Implementation of telehealth consultation modalities for adolescent health.** |
| 75 Tavener, C. R. et al. | **Implementation of virtual consultations for antenatal care.** |
| 76 Phillips, T. R. et al. | **Various changes in service delivery in Australian public sexual health clinics.** |
| 77 Farrell, R. et al. | **Rapid implementation of telehealth modalities for obstetrics, supplemented by self-monitoring components.** |
| 78 Chong, E. et al. | **Implementation of a direct-to-patient telemedicine abortion service called the "TelAbortion Project", which is a direct-to-patient service model whereby participating clinics counsel and screen patients remotely, and then send mifepristone and misoprostol by mail to those who are eligible.** |
| 79 Stifani, B. M. et al. | **Rapid implementation of telehealth for contraceptive counseling.** |
