# Supplementary material for: Sexual and reproductive health service delivery innovations and adaptations during COVID-19: A systematic review and crowdsourcing open call
Source: PLOS Glob Public Health. 2025 Sep 10;5(9):e0002032. doi: 10.1371/journal.pgph.0002032 (PMC12422507; doi:10.1371/journal.pgph.0002032)
Supplement: S4 Table — (DOCX) [file pgph.0002032.s004.docx]

**S4 Table: Open call submissions contributing to the study findings**

| **S/N** | **Submission Title** | **Submission URL** |
| --- | --- | --- |
| 1 | Expanding Access To Safe, Medical Abortion During The  COVID-19 Pandemic With Telemedicine In England And South Africa | Https://Docs.Google.Com/Document/D/1kas9sy3n6ru kwbv3mrwzou3abiz6ekam/Edit |
| 2 | Telemedicine For Abortion Provision | Https://Drive.Google.Com/File/D/16l-  F2ya3dazmul9cjzuli8v6ebfudjkb/View |
| 3 | Using Mobile Application For Continued Access To HIV  Services Among Key Populations: An COVID-19 Adaptation In Namibia | Https://Docs.Google.Com/Document/D/1dlwdkxb0r6g alopmbedduvw7wj6tpfkn/Edit |
| 4 | Fighting Gender-Based Violence During COVID-19: A  Success Story In Zimbabwe | Https://Docs.Google.Com/Document/D/1V T6zunvt  brenoeobdwioo4owcficew/Edit |
| 5 | How PSI's India Private Limited Is Providing SRHR Products And Services During A Pandemic | Https://Docs.Google.Com/Document/D/1eynmidqauicj or2k_V7ktjttlegi4ilh/Edit |
| 6 | ACHIEVE And Usaids Partnerships To Reduce HIV/AIDS In Adolescent Girls And Young Women (AGYW) During COVID-19 | Https://Docs.Google.Com/Document/D/1pxyh8e_E9y- Npllxucz5-Ngtae3nvlkm/Edit |
| 7 | Tanzania Maternal And New Born Health During COVID-  19 (UNHCR). Fighting Gender-based Violence during COVID-19 A Success Story in Zimbabwe | Https://Docs.Google.Com/Document/D/1t82_Vkkp- Gi7eto18vn1y3xezkeyn62q/Edit |
| 8 | Leveraging Community-Based Family Planning Distributors Amidst The Covid-19 Pandemic To Increase Access And Utilization Of Contraception By Adolescent Girls In Northern Uganda | Https://Docs.Google.Com/Document/D/1qqvzs72rm1q xnkmrfuz19omt6d6vyknd/Edit |
| 9 | UNFPA APRO And Burnet Institute Australia Submission | Https://Docs.Google.Com/Document/D/1u_Outpdpwb gljggbbiteat7celr20kqp/Edit |
| 10 | WHOHRP Call | Https://Docs.Google.Com/Document/D/1zcuzzn6kxrrs  vpz5hzwow6uwkgnnq6sk/Edit |
| 11 | Changes in ASRH services during covid 19 Ingobyi Activity Rwanda.docx | https://docs.google.com/document/d/19UnZgVui_eSC StEaTieHvTuzSj8qB8HA/edit |
| 12 | A New Referral System Connects More Women And Girls In Eastern Uganda To  Family Planning | Https://Drive.Google.Com/File/D/1aisawhnuum- Q4tsohqfrlprrlamdr1cq/View |
| 13 | ATLAS_WHO Open call SRH-COVID (FRENCH) | Https://Drive.Google.Com/File/D/16eo_L968dpayb12t  cjbahm4n-Tbhvh2-/View |
| 14 | Digital Tools To Deliver Adolescents And Youth Sexual And Reproductive Health Messages (AYSRH) During  COVID-19 Pandemic | Https://Docs.Google.Com/Document/D/19unzgvui_Es csteatiehvtuzsj8qb8ha/Edit |
| 15 | Togo COVID-19 IPPF Innovation And Best Practice -  Telemedicine Abortion Care. | Https://Drive.Google.Com/File/D/1jz75suoyohbpdc0q  em788uk7m3skusxh/View |
| 16 | COVID YEP WHO | https://drive.google.com/drive/folders/1jSyptPVYKmo  JAdi3fpCdasv2Li-e7Set |
| 17 | DAScreeningInPLWHWHOOpemCall23.2.22 | https://docs.google.com/document/d/10MZr82jK0D7Q lEBrWtOkDDwGaxR5YAqu_nwa3SYyTys/edit |
| 18 | Service adaptations to HIV prevention interventions in  Bangladesh | https://docs.google.com/document/d/1rzJ1qjkEeZZUJ  OEAfEcqVzOyw5CRrIEWJ8VZbhUgi3E/edit |
